# Effects of Baclofen on Central Paroxysmal Positional Downbeat Nystagmus

**DOI:** 10.1007/s12311-024-01684-z

**Published:** 2024-03-18

**Authors:** So-Yeon Yun, Jong-Hee Lee, Hyo-Jung Kim, Jeong‑Yoon Choi, Ji-Soo Kim

**Affiliations:** 1 Present Address: Department of Neurology, Severance Hospital, Yonsei University College of Medicine, Seoul, Republic of Korea; 2 Dizziness Center, Seoul National University Bundang Hospital, Seongnam, Republic of Korea; 3 Biomedical Research Institute, Seoul National University Bundang Hospital, Seongnam, Republic of Korea; 4 Present Address: Department of Neurology, Seoul National University College of Medicine, Seoul, Republic of Korea

**Keywords:** Vertigo, Central positional nystagmus, Baclofen

## Abstract

Paroxysmal positional nystagmus frequently occurs in lesions involving the cerebellum, and has been ascribed to disinhibition and enhanced canal signals during positioning due to cerebellar dysfunction. This study aims to elucidate the mechanism of central positional nystagmus (CPN) by determining the effects of baclofen on the intensity of paroxysmal positional downbeat nystagmus due to central lesions. Fifteen patients with paroxysmal downbeat CPN were subjected to manual straight head-hanging before administration of baclofen, while taking baclofen 30 mg per day for at least one week, and two weeks after discontinuation of baclofen. The maximum slow phase velocity (SPV) and time constant (TC) of the induced paroxysmal downbeat CPN were analyzed. The positional vertigo was evaluated using an 11-point numerical rating scale (0 to 10) in 9 patients. After treatment with baclofen, the median of the maximum SPV of paroxysmal downbeat CPN decreased from 30.1°/s [interquartile range (IQR) = 19.6—39.0°/s] to 15.2°/s (IQR = 11.2—22.0°/s, Wilcoxon signed rank test, *p* < 0.001) with the median decrement ratio at 40.2% (IQR = 28.2—50.6%). After discontinuation of baclofen, the maximum SPV re-increased to 24.6°/s (IQR = 13.1—34.4°/s, Wilcoxon signed rank test, *p* = 0.001) with the median increment ratio at 23.5% (IQR = 5.2—87.9%). In contrast, the TCs of paroxysmal downbeat CPN remained unchanged at approximately 3.0 s throughout the evaluation. The positional vertigo also decreased with the medication (Wilcoxon signed rank test, *p* = 0.020)*,* and remained unchanged even after discontinuation of medication (Wilcoxon signed rank test, *p* = 0.737). The results of this study support the prior presumption that paroxysmal CPN is caused by enhanced responses of the semicircular canals during positioning due to cerebellar disinhibition. Baclofen may be tried in symptomatic patients with paroxysmal CPN.

**Supplementary Information:**

The online version contains supplementary material available at 10.1007/s12311-024-01684-z.

## Introduction

Positional nystagmus refers to the nystagmus triggered by the changes in the dependent position of the head relative to gravity [1, 2]. According to its temporal characteristics, positional nystagmus can be divided into paroxysmal or persistent type. Either paroxysmal or persistent type of positional nystagmus may occur in central as well as peripheral vestibular lesions. Central positional nystagmus (CPN) mostly presents with apogeotropic nystagmus in the ear-down position or downbeat nystagmus after lying down or straight head-hanging (SHH) [3]. In previous studies, the paroxysmal CPN has been ascribed to disinhibition and enhanced canal signals during positioning due to cerebellar dysfunction [2, 4]. Indeed, the paroxysmal CPN mostly occurs in the planes of semicircular canals that are inhibited during the positioning, and shows the features suggestive of a semicircular canal origin regarding the latency and duration [2]. Furthermore, the intensity of paroxysmal downbeat CPN induced during SHH depends on the positioning head velocity [4]. Since the lesions having caused paroxysmal CPN were mostly overlapped in the cerebellar nodulus and uvula, disruption of GABAergic inhibition over the velocity-storage mechanism (VSM) may be presumed as the mechanism of paroxysmal CPN [2, 5]. Previously, the effect of baclofen, a GABA_B_ receptor agonist, was evaluated in acquired pendular nystagmus and spontaneous vertical nystagmus [5, 6]. This study aims to elucidate the mechanism of CPN by determining the effects of baclofen on the intensity of paroxysmal positional downbeat nystagmus due to central lesions. We hypothesized that the intensity of induced nystagmus would decrease with the administration of baclofen and subsequently increase upon its discontinuation.

## Materials and Methods

### Patients

We had recruited 15 patients (11 women, mean age ± SD = 55.7 ± 13.0 years, age range = 23—73 years) with paroxysmal downbeat CPN, irrespective of the presence of positional vertigo, at the Dizziness Center of Seoul National University Bundang Hospital from September 2020 to December 2022. The median duration from the symptom (dizziness or unsteadiness) onset to evaluation was 5.0 years (IQR = 2.5—10 years, range = 0.3—13 years, Table 1). All patients underwent detailed neuro-otologic evaluation by the senior author (J.S.K). The diagnosis of paroxysmal downbeat CPN was based on (1) paroxysmal downbeat nystagmus (< 1 min) induced during SHH, (2) presence of other symptoms and signs indicative of brainstem or cerebellar dysfunction, or brainstem or cerebellar lesions documented on MRIs, and (3) no resolution of paroxysmal downbeat nystagmus with two or more application of reverse Epley or Yacovino maneuver for anterior canal benign paroxysmal positional vertigo involving the anterior semicircular canals. All patients were subjected to manual straight head-hanging (SHH) at baseline (before medication of baclofen), while taking baclofen 30 mg per day for at least one week, and two weeks after discontinuation of baclofen (Supplement Table 1). The patients themselves and their caregivers decided to participate in the study after being comprehensively informed about the potential efficacy and adverse effects of baclofen. Information was collected on demographic characteristics including age and sex, and concomitant medications such as clonazepam, gabapentin and 3–4-diaminopyridine, and the primary diagnosis from the electronic medical records.

**Table 1:** Demographic and clinical characteristics of the patients

Patient	Etiology	Duration of symptoms (years)	Symptoms					Medication other than baclofen
			Vertigo	Gait ataxia	Dysarthria	Oscillopsia	Diplopia	
1	Cerebellar ataxia, unknown etiology	2	+	+	-	+	-	none
2	Multiple systemic atrophy	2	+	+	+	-	-	betahistine, choline alfoscerate, midrone
3	Spinocerebellar ataxia, type 17	3	+	+	+	-	+	betahistine, choline alfoscerate
4	Episodic ataxia, type 2	11	+	+	-	-	-	betahistine, acetazolamide
5	Multiple systemic atrophy	3	+	+	-	-	-	betahistine, oxiracetam, escitalopram
6	Spinocerebellar ataxia, type 1	5	+	+	-	+	+	acetazolamide, choline alfoscerate
7	Chiari malformation	10	+	+	+	+	-	none
8	Cerebellar ataxia, unknown etiology	5	+	-	-	-	-	none
9	Multiple systemic atrophy	3	+	+	+	-	-	none
10	Spinocerebellar ataxia, type 6	13	+	+	+	-	+	choline alfoscerate, nilotinib
11	Multiple systemic atrophy	1	+	+	+	-	-	pyridostigmine, escitalopram
12	Spinocerebellar ataxia	13	+	+	+	+	+	none
13	Spinocerebellar ataxia	8	+	+	-	+	+	acetazolamide
14	Cerebellar ataxia, unknown etiology	10	+	-	-	-	-	alprazolam, propanolol
15	Paraneoplastic cerebellar degeneration	0.3	+	+	+	+	-	none

+  = present; - = absent

### Oculography

Eye movements were recorded binocularly at a sampling rate of 120 Hz using 3-dimensional video-oculography (VOG, SLVNG, SLMED, Seoul, South Korea). Spontaneous nystagmus was recorded both with and without visual fixation in the sitting position. Gaze-evoked nystagmus, horizontal smooth pursuit, horizontal saccades, and horizontal head-shaking nystagmus were also evaluated.

### Positioning maneuvers

To ensure the effect of baclofen, the recording conditions had been controlled throughout the evaluation [7–9]. First, the recordings were arranged at a similar time of the day after maintaining upright position at least for one hour before the recording (Supplement Table 1). During the recording, the patients were instructed to maintain the straight ahead gaze, which was also monitored on the screen and recording traces. During the SHH, patients were laid from the sitting upright position onto the lying down position with the head extended about 30° below the table. This positioning was completed usually within 3 s (2—4 s), resulting in an average head rotation velocity of an approximately 40^°^/s during the positioning [4]. In each testing condition, the SHH position was maintained until the positional nystagmus disappeared or at least for one minute. The positional vertigo was assessed using an 11-point numerical rating scale (0 to 10) in 9 patients.

### Analyses of Nystagmus

Digitized eye position data were analyzed with MATLAB software (version R2019b, The Math Works, Inc., MA USA). For each paroxysmal downbeat CPN, the intensity (maximum slow phase velocity, SPV) and time constant (TC) were calculated. When persistent CPN was combined with the paroxysmal one, we calculated the maximum SPV and TC of paroxysmal CPN after subtracting the persistent component from the velocity profile of induced positional nystagmus [2].

### Statistical Analyses

The categorical variables are presented as the numbers and percentages, continuous variables conforming to a normal distribution as the mean ± standard deviation (SD), and the variables that are not normally distributed as the median and interquartile range (IQR). Normality of the data was determined using the Shapiro–Wilk test. Changes in the maximum SPV and TC of paroxysmal downbeat CPN and the positional vertigo after the baclofen treatment, and after discontinuation of baclofen were assessed using the Wilcoxon signed rank test. The Spearman correlation test was used to compare the maximum SPV of paroxysmal downbeat CPN with the severity of positional vertigo. The Mann–Whitney U test was used to analyze any associations of the clinical factors with the responsiveness to baclofen. For all analyses, a two-tailed *p* value of < 0.05 was considered statistically significant. All statistical analyses were performed using the R software package (version 4.3.1).

## Results

### Clinical Characteristics

Underlying etiologies of paroxysmal downbeat CPN included spinocerebellar ataxia (n = 5, 33.3%), multiple systemic atrophy (n = 4, 26.7%), episodic ataxia (n = 1, 6.7%), Chiari malformation (n = 1, 6.7%) and paraneoplastic cerebellar degeneration (n = 1, 6.7%). No etiology was identified in the remaining three patients (Table 1). Patients had dizziness (n = 15, 100%), gait ataxia (n = 13, 86.7%) and dysarthria (n = 8, 53.3%). During visual fixation, four patients (26.7%) showed spontaneous nystagmus that was pure downbeat in two, pure upbeat in one and mixed horizontal-downbeat in the remaining one. Without visual fixation in darkness, 10 patients (66.7%) showed spontaneous nystagmus that was pure downbeat in four, pure upbeat in three, pure horizontal in two, and mixed horizontal-downbeat in the remaining one. After horizontal head-shaking, 14 patients (93.3%) showed nystagmus that was mixed horizontal-downbeat in seven, pure downbeat in five, and pure horizontal in two. Ten patients (66.7%) showed gaze-evoked nystagmus, and four of them also had rebound nystagmus (Table 2). Horizontal smooth pursuit was impaired in 14 patients (93.3%). Horizontal saccades were abnormal in 12 patients (80.0%), hypermetric in 10 and hypometric in two.

**Table 2:** Ocular motor findings in the patients

Pt	SN, fixation	SN, non- fixation	GEN	HSN	maximum SPV (°/s)			TC (s)		
					Baseline	Treatment with baclofen	After discontinuation	Baseline	Treatment with baclofen	After discontinuation
1	-	D	+	L + D + CCW	61.2	48.2	50.3	5.4	3.2	2.8
2	-	U	+ (REB)	R	32.1	15.9	30.8	2.2	1.4	2.0
3	-	R	+ (REB)	R + D → L + D	27.2	13.4	15.5	1.4	1.1	2.8
4	-	R + D	+	L + D	29.8	20.7	37.7	2.0	3.0	2.5
5	-	-	-	L + D	16.3	12.1	12.0	8.0	8.5	6.8
6	-	D	+ (REB)	D	68.1	40.7	67.6	1.7	1.7	2.2
7	R + D	R	+ (REB)	R	40.4	10.8	31.0	2.3	2.1	2.6
8	-	-	-	-	37.9	23.2	24.6	2.0	1.9	2.1
9	-	-	-	D	20.8	11.5	14.5	4.1	3.5	1.2
10	-	-	-	D	35.3	10.2	12.6	1.3	2.3	1.7
11	-	-	-	D	13.9	17.1	13.5	7.8	3.2	8.8
12	D	D	+	R + D	18.3	5.4	12.6	1.8	1.1	1.7
13	D	U	+	D → U	30.1	15.2	39.9	5.0	2.0	4.5
14	-	D	-	R + D	7.7	7.6	9.0	3.6	3.8	2.3
15	U	U	-	L + D → U	40.1	26.3	26.7	1.6	1.7	1.9

*D* = Downbeat nystagmus; GEN = Gaze-evoked nystagmus; HSN = Head shaking nystagmus; L = Left beating nystagmus; C = Cerebellum; P = Pontine; *M* = Middle cerebellar peduncle; *R* = Right beating nystagmus; REB = Rebound; SN = Spontaneous nystagmus; SPV = Slow phase velocity; TC = Time Constant; *U* = Upbeat nystagmus; +  = present; - = absent

During the SHH, the downbeat CPN was pure paroxysmal in 13 and mixed paroxysmal and persistent in two patients. The paroxysmal downbeat CPN reached a peak within a few seconds (median = 1.1 s, IQR = 0.8–2.8 s). After then, the nystagmus decreased exponentially.

When recruited, some patients were taking medications such as betahistine, acetazolamide, and escitalopram, which had been maintained throughout the evaluation without a change in dosage (Table 1).

### Effects of Baclofen on Paroxysmal Downbeat CPN

**Fig. 1:**
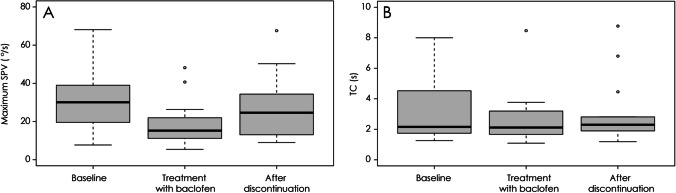
Effects of baclofen on paroxysmal downbeat central positional nystagmus (CPN). (**A**) Baclofen administration resulted in a significant reduction of the maximum slow phase velocity (SPV) of paroxysmal downbeat CPN. After discontinuation of baclofen, the maximum SPV re-increased significantly. (**B**) The time constants of paroxysmal downbeat CPN remained unchanged throughout the evaluation

**Fig. 2:**
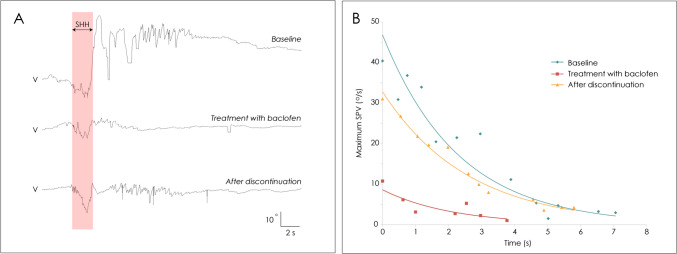
Representative recording of paroxysmal downbeat nystagmus induced during straight head-hanging in patient 7 with Chiari malformation. (**A**) Video-oculographic recording of eye movements shows paroxysmal downbeat positional nystagmus at baseline, during administration of baclofen, and after discontinuation of baclofen. V: vertical position of the left eye. Upward deflection denotes upward eye motion in each recording. The shaded areas in pink indicate the period of positioning. (**B**) Vertical slow phase velocity of the left eye in each recording. After administration of baclofen for two weeks, the maximum slow phase velocity (SPV) of induced paroxysmal downbeat nystagmus decreased from 40.4 to 10.8°/s. The maximum SPV re-increased to 31.0°/s 2 weeks after discontinuation of baclofen. In contrast, the time constants of induced paroxysmal downbeat nystagmus remained unchanged throughout the evaluation

At baseline, the maximum SPV of paroxysmal downbeat CPN ranged from 7.7 to 68.1 (median = 30.1°/s, IQR = 19.6—39.0°/s). After medication of baclofen for 2–4 weeks, the maximum SPV was measured from 5.4 to 48.2 (median = 15.2°/s, IQR = 11.2—22.0°/s) with a significant decrease in the intensity (Wilcoxon signed rank test, *p* < 0.001). The decrement was calculated from 23.0 to 73.3% (median = 40.2%, IQR = 28.2—50.6%). After discontinuation of baclofen, the paroxysmal downbeat CPN re-increased with the median maximum SPV at 24.6°/s (IQR = 13.1—34.4°/s, Wilcoxon signed rank test, *p* = 0.001) and the median increment at 23.5% (IQR = 5.2—87.9%) **(**Figs. 1A and 2, Supplement Video). In contrast, the TCs of paroxysmal downbeat CPN remained unchanged at approximately 3.0 s throughout the evaluation (Fig. 1B).

**Fig. 3:**
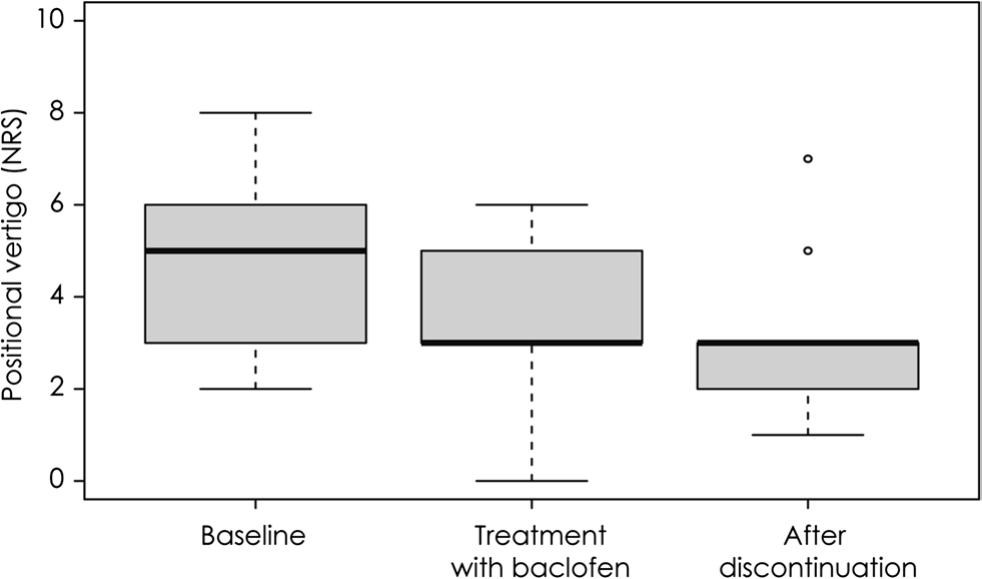
Changes in the positional vertigo. The positional vertigo decreased significantly with medication of baclofen and the reduction of positional vertigo persisted even after discontinuation of medication. NRS = Numerical rating scale (0—10)

The positional vertigo also decreased with medication (Wilcoxon signed rank test, *p* = 0.020), and remained unchanged even after discontinuation of medication (Wilcoxon signed rank test, *p* = 0.737, Fig. 3). The severity of positional vertigo did not correlate with the maximum SPV of paroxysmal downbeat CPN among the patients (Spearman correlation test, *p* = 0.810).

One patient (patient 10) had perioral tightness with baclofen. Univariate analyses showed no significant associations between decrements of the maximum SPV after baclofen administration and the age and sex of the patients, underlying etiology, and the duration from symptom onset to baclofen trial.

## Discussion

This study documented a decrease in the maximum SPV of paroxysmal downbeat CPN with administration of baclofen, a GABA_B_ agonist. This result supports our prior presumption that the paroxysmal CPN results from disinhibited and abnormally enhanced canal responses during positioning due to cerebellar dysfunction [2, 4].

The characteristics of paroxysmal downbeat CPN observed in this study are consistent with those of prior studies regarding its peak at onset, short duration with a TC around 3.0 s, and alignment of the nystagmus direction with the vector sum of the rotational axes of the semicircular canals that are normally inhibited during the positioning [2]. Given the lesions responsible for paroxysmal CPN located in the cerebellum, especially the nodulus and uvula, impaired cerebellar inhibition over the vestibular system is plausible as a mechanism of paroxysmal CPN [2, 10]. The irregular type of primary vestibular afferents is responsible for adaptation and the VSM of the vestibulo-ocular reflex [11, 12]. This adaptation is characterized by a rapid decline in the discharges and a rebound when the discharges go below the resting rate. And then, the discharges gradually return to the resting level. These adaptive responses are termed as the post-acceleratory secondary phenomenon [13]. The VSM plays a role in estimating the gravitational direction by integrating the rotation cues from the semicircular canal signals [14, 15]. There is a rotational feedback loop within the VSM, which acts to adjust the estimated gravitational direction to the real one. Therefore, lesions involving the vestibulocerebellum would lead to an exaggerated post-acceleratory secondary response and post-rotatory nystagmus provoked by a difference between the estimated and real gravitational directions [2, 14]. Thus, CPN may be ascribed to enhanced secondary phenomenon of the irregular afferents due to lesions involving the nodulus and uvula [2]. In our previous study, the intensity of paroxysmal downbeat CPN induced during SHH depended on the positioning head velocity [4]. This also supports that paroxysmal downbeat CPN is generated by the canal-driven signals during the positioning maneuvers.

Baclofen is a GABA_B_ receptor agonist that enhances the inhibitory action of the vestibulocerebellum on the vestibular nuclei, and particularly inhibits the VSM of the vestibulo-ocular reflex [5, 16]. Indeed, the action of baclofen has been demonstrated by effectively inhibiting periodic alternating nystagmus, which is also ascribed to enhanced VSM due to loss of inhibition from the cerebellar nodulus and uvula [17, 18]. In patients with an acute lateral medullary infarction, baclofen suppressed head-shaking nystagmus that is also explained by asymmetry of the VSM due to unilateral interruption of the cerebellar inhibitory inputs [19]. Furthermore, baclofen can also reduce acquired spontaneous downbeat nystagmus and vertical pendular nystagmus [5, 6]. Given this background, we investigated the effects of baclofen on the intensity of paroxysmal positional downbeat nystagmus due to central lesions. This was also to ascertain our previous hypothesis on the mechanism of CPN, abnormally enhanced canal responses during positioning due to cerebellar disinhibition. Indeed, we found that baclofen reduces the intensity of paroxysmal downbeat CPN during SHH.

Notably, the reduction in the intensity of paroxysmal downbeat nystagmus after baclofen administration was not related to the underlying etiology of CPN, although the response was most pronounced in patients with spinocerebellar ataxia (SCA). Considering that all five patients with SCA showed a marked reduction (> 50%) of nystagmus, the association between the underlying etiology and baclofen effect might have been insufficiently demonstrated due to the small study population.

After administration of baclofen, the positional vertigo decreased along with reduction of positional nystagmus. However, the improved symptom persisted even after discontinuation of medication and re-increase of positional nystagmus. These findings again indicate that the severity of positional vertigo does not correlate with the intensity of positional nystagmus in central lesions, a well-established finding from the previous studies [1, 20, 21]. Otherwise, the 11-point numerical rating scale adopted in this study for grading the vertigo severity could not accurately reflect the subjective symptoms.

There are several limitations in our study. First, because of the small sample size, we were unable to perform multivariate analyses to evaluate the factors associated with the efficacy of baclofen. Second, we only quantified paroxysmal downbeat CPN, and did not include other types of paroxysmal CPN such as geotropic or apogeotropic horizontal nystagmus during head turning to either side while supine. Third, we did not consider apogeotropic benign paroxysmal positional vertigo involving the posterior canal as a rare cause of paroxysmal positional downbeat nystagmus. Finally, the long-term effect of baclofen was not evaluated in this study.

## Conclusion

This study supports the prior presumption that paroxysmal CPN is caused by enhanced responses of the semicircular canals during positioning due to cerebellar disinhibition. Baclofen may be a safe and effective treatment option for patients with symptoms due to paroxysmal downbeat CPN regardless of the underlying etiologies. Future clinical trials involving a larger number of patients are required to validate the efficacy of baclofen and identify the optimal dose and duration of medication.

## Supplementary Information

Below is the link to the electronic supplementary material.

Supplementary file1 (DOCX 18 KB)

Supplementary file2 (MP4 115860 KB) Straight head-hanging induced paroxysmal downbeat central positional nystagmus in patient 7 with Chiari malformation. At baseline (before administration of baclofen) → During administration of baclofen for two weeks → two weeks after discontinuation of baclofen

## Data Availability

Anonymized data will be shared upon request form any qualified investigator.
