# Supplementary material for: Effects of Baclofen on Central Paroxysmal Positional Downbeat Nystagmus
Source: Cerebellum. 2024 Mar 18;23(5):1892–8. doi: 10.1007/s12311-024-01684-z (PMC11489365; doi:10.1007/s12311-024-01684-z)
Supplement: Supplementary file 1 — Supplementary file1 (DOCX 18 KB) [file 12311_2024_1684_MOESM1_ESM.docx]

Supplement Table 1. Treatment and follow-up profiles of the patients

| Patient | Baclofen dose, duration | Duration of baclofen administration  (day) | Duration after baclofen discontinuation (day) | SHH test time | | |  |
| --- | --- | --- | --- | --- | --- | --- | --- |
|  |  |  |  | Baseline | During administration | After discontinuation |  |
| 1 | 15 mg/d, 7 days → 30 mg/d, 7 days | 15 | 14 | 13:40 | 13:40 | 13:40 |  |
| 2 | 15 mg/d, 7 days → 30 mg/d, 7 days | 14 | 14 | 11:40 | 11:40 | 11:30 |  |
| 3 | 15 mg/d, 7 days → 30 mg/d, 7 days | 25 | 16 | 11:30 | 11:30 | 11:30 |  |
| 4 | 15 mg/d, 7 days → 30 mg/d, 7 days | 14 | 15 | 16:30 | 16:30 | 16:30 |  |
| 5 | 15 mg/d, 7 days → 30 mg/d, 7 days | 14 | 13 | 13:40 | 13:40 | 13:40 |  |
| 6 | 15 mg/d, 7 days → 30 mg/d, 21 days | 28 | 33 | 12:00 | 12:00 | 12:00 |  |
| 7 | 15 mg/d, 7 days → 30 mg/d, 7 days | 14 | 14 | 12:00 | 12:00 | 12:00 |  |
| 8 | 15 mg/d, 7 days → 30 mg/d, 7 days | 14 | 14 | 12:00 | 12:00 | 12:00 |  |
| 9 | 15 mg/d, 7 days → 30 mg/d, 7 days | 14 | 14 | 9:30 | 9:30 | 9:30 |  |
| 10 | 15 mg/d, 7 days → 30 mg/d, 7 days | 23 | 15 | 11:00 | 11:00 | 10:00 |  |
| 11 | 15 mg/d, 7 days → 30 mg/d, 7 days | 19 | 15 | 13:50 | 11:40 | 11:10 |  |
| 12 | 15 mg/d, 7 days → 30 mg/d, 7 days | 14 | 14 | 10:00 | 10:00 | 10:00 |  |
| 13 | 15 mg/d, 7 days → 30 mg/d, 7 days | 15 | 13 | 10:00 | 10:00 | 10:00 |  |
| 14 | 15 mg/d, 7 days → 30 mg/d, 7 days | 13 | 15 | 14:20 | 14:20 | 14:20 |  |
| 15 | 15 mg/d, 7 days → 30 mg/d, 7 days | 14 | 14 | 11:30 | 11:00 | 11:00 |  |
